# Suppressing of Src–Hic-5–JNK–AKT Signaling Reduced GAPDH Expression for Preventing the Progression of HuCCT1 Cholangiocarcinoma

**DOI:** 10.3390/pharmaceutics14122698

**Published:** 2022-12-02

**Authors:** Wen-Sheng Wu, Rui-Fang Chen, Chuan-Chu Cheng, Jia-Ling Wei, Chen-Fang Lin, Ren-In You, Yen-Chang Chen, Ming-Che Lee, Yen-Cheng Chen

**Affiliations:** 1 Division of General Surgery, Department of Surgery, Hualien Tzu Chi Hospital, Buddhist Tzu Chi Medical Foundation, Hualien 970, Taiwan; 2 Institute of Medical Sciences, Tzu Chi University, Hualien 970, Taiwan; 3 Department of Laboratory Medicine and Biotechnology, College of Medicine, Tzu Chi University, Hualien 970, Taiwan; 4 Department of Anatomical Pathology, Hualien Tzu Chi Hospital, Buddhist Tzu Chi Medical Foundation, Hualien 970, Taiwan; 5 Department of Pathology, School of Medicine, Tzu Chi University, Hualien 970, Taiwan; 6 Division of General Surgery, Department of Surgery, Wan Fang Hospital, Taipei Medical University, Taipei 110, Taiwan; 7 Department of Surgery, School of Medicine, College of Medicine, Taipei Medical University, Taipei 110, Taiwan; 8 School of Medicine, Tzu Chi University, Hualien 970, Taiwan

**Keywords:** cholangiocarcinoma, HuCCT1, TFK1, hydrogen peroxide clone-5, Src nonreceptor tyrosine kinase, glyceraldehyde-3-phosphate dehydrogenase

## Abstract

Cholangiocarcinoma (CCA) is a malignant neoplasm of the bile ducts, being the second most common type of cancer in the liver, and most patients are diagnosed at a late stage with poor prognosis. Targeted therapy aiming at receptors tyrosine kinases (RTKs) such as c-Met or EGFR have been developed but with unsatisfactory outcomes. In our recent report, we found several oncogenic molecules downstream of RTKs, including hydrogen peroxide clone-5 (Hic-5), Src, AKT and JNK, were elevated in tissues of a significant portion of metastatic CCAs. By inhibitor studies and a knockdown approach, these molecules were found to be within the same signal cascade responsible for the migration of HuCCT1 cells, a conventionally used CCA cell line. Herein, we also found Src inhibitor dasatinib and Hic-5 siRNA corporately suppressed HuCCT1 cell invasion. Moreover, dasatinib inhibited the progression of the HuCCT1 tumor on SCID mice skin coupled with decreasing the expression of Hic-5 and EGFR and the activities of Src, AKT and JNK. In addition, we found a glycolytic enzyme glyceraldehyde-3-phosphate dehydrogenase (GAPDH) and several cytoskeletal molecules such as tubulin and cofilin were dramatically decreased after a long-term treatment of the HuCCT1 tumor with a high dose of dasatinib. Specifically, GAPDH was shown to be a downstream effector of the Hic-5/Src/AKT cascade involved in HuCCT1 cell migration. On the other hand, TFK1, another CCA cell line without Hic-5 expression, exhibited very low motility, whereas an ectopic Hic-5 expression enhanced the activation of Src and AKT and marginally increased TFK1 migration. In the future, it is tempting to investigate whether cotargeting Src, Hic-5 and/or GAPDH is efficient for preventing CCA progression in future clinical trials.

## 1. Introduction

Cholangiocarcinoma (CCA) is the second most common primary liver cancer [1,2] which is the most prevalent in Southeast Asia (as high as 80/100,000 incidence rate) [3]. CCA originates from the epithelium lining the biliary tree and is anatomically classified as intrahepatic CCA (iCCA, proximal to the second order bile ducts), perihilar CCA (p-CCA, between the second order bile ducts and the insertion of the cystic duct) and distal extrahepatic CCA (dCCA, between the insertion of the cystic duct and the ampulla of Vater) [4,5,6]. Previously, the incidence rate of iCCAs was less than that of p-CCA and eCCA; however, it has been increasing while the other two’s has been decreasing in recent years [7,8,9]. There are different molecular characteristics and biology between these subtypes of CCAs, which need to be treated by distinct therapeutic approaches [6]. CCA is an aggressive and malignant cancer, and most patients are diagnosed at a late stage with a median survival of less than 24 months [10,11]. While curative resection for CCA in combination with adjuvant chemotherapy (5-fluoruracil, gemcitabine, oxaliplatin, etc.) is effective only in early stages, systemic therapies is the single option for unresectable cases with poor outcome. Moreover, recurrence rate is high after surgery of CCA within 5 years [12,13], which is closely associated with microvascular invasion and lymph node metastases [14,15]. Thus, it is an unmet need to develop effective strategies such as targeted therapy to prevent CCA metastasis. 

Tumor metastasis is initiated by an epithelial–mesenchymal transition (EMT), migration and invasion followed by intravasation, extravasation and final invasion on metastatic loci [16,17]. A lot of growth factors and cytokines in a tumor microenvironment can trigger the critical steps in metastasis. The pathological mechanisms for CCA progression have been investigated in the past decades [18,19]. It was recently suggested that chronic liver diseases including primary sclerosing cholangitis (PSC) [20], cirrhosis, biliary stone and certain bacterial, viral or parasitic infections all increased the risks of CCA due to chronic inflammation or cholestasis (for review 4). While inflammation increased the exposure of cholangiocytes to cytokines such as interleukin-6 [21] and Wnt [22], cholestasis leads to the overexposure of cholangiocytes to bile acids [23] that cause abnormal cell proliferation and cholangiocarcinogenesis [24]. Moreover, intimate interactions between extracellular cytokines and overexpressed surface receptors trigger dysregulated intracellular pathways promoting cell proliferation migration and invasion. Indeed, the cell surface receptor tyrosine kinases (RTKs) such as c-Met, Erb family EGFR, Her2 and Her3 along with their cognate ligands are abnormally high in CCAs [4,25,26]. Specifically, 62% of iCCAs with uncontrolled progression were characterized by the activation of c-Met, EGFR and downstream mitogen-activated protein kinase (MAPK) signaling [27]. Nonetheless, inhibitory drugs that can target cell surface receptors such as c-Met and EGFR [28,29,30] or the components in the MAPK cascade such as MEK [31] were unsatisfactory in clinical trials for preventing CCA progression [5]. In fact, targeting metastatic signaling such as oncogenic RTKs often suffered from acquired or inducible drug resistances [32], largely due to a co-expression of multiple RTKs that can raise compensatory secondary signaling.

Another issue for molecular targeted therapy regards precision medicine. CCA represents a heterogeneous tumor with diverse molecular alterations [4] implying that the signal pathways responsible for triggering CCA progression might be different between individual patients. Thus, it is essential to screen the actual signaling pathway deregulated in clinical CCA samples, which can help to identify the feasible targets for preventing CCA progression in a personalized manner.

In our recent report [33], we screened the deregulated signaling in the surgical tissues of CCA patients and found the activities and/or expression of several oncogenic molecules downstream of RTKs including hydrogen peroxide clone5 (Hic-5), known to be a critical mediator of hepatocellular carcinoma (HCC) progression [34,35]; Src, one of the nonreceptor tyrosine kinase; AKT, the serine threonine kinase downstream of PI3K; and JNK, one of the MAPK, elevated in tissues of a significant portion (about 40–50%) of metastatic CCAs. These molecules were demonstrated to be within the same signaling pathway responsible for the migration of HuCCT1 cells, a conventionally used iCCA cell line [33]. It is worth noting that not only the roles of Hic-5, Src, AKT and JNK in metastatic signaling have been well established, but also the interaction and regulation between them have been frequently mentioned. For example, Src activity was required for Hic-5 to promote TGF-β-induced EMT [36] and for AKT-mediated EMT and the migration of gastric cancer [37]. Furthermore, JNK positively cross-talks with Hic-5 in HCC progression [35]. In the current study, we further validated that the Hic-5/Src/AKT/JNK cascade was also associated with HuCCT1 cell invasion and progression in SCID mice. In addition, we found several cytoskeletal molecules, including tubulin [38], cofilin [39] and a glycolytic enzyme glyceraldehyde-3-phosphate dehydrogenase (GAPDH) known to be involved in tumor progression, were dramatically decreased after a long-term treatment of the HuCCT1 tumor on mouse skin with the Src inhibitor dasatinib. Specifically, GAPDH gene expression was also suppressed by dasatinib in cultured HuCCT1 cells and shown to be a downstream effector of the Hic-5/Src/AKT cascade involved in HuCCT1 cell migration in vitro. 

## 2. Materials and Methods

### 2.1. Cell Culture

HuCCT1, a human bile duct carcinoma cell line with metastatic ability, and TFK-1, established from a surgical tumor of extrahepatic bile duct carcinomas, were originally obtained from Cell Bank in RIKEN BioResource Research Center (Ibaraki, Japan). The cells were cultured in RPMI1640 with 10% FBS kept at 37 °C, 5% CO_2_.

### 2.2. Antibodies and Chemicals

Rabbit polyclonal antibodies of Hic-5, phosphorylated Src (p-Src), and AKT (p-AKT) and Ki67 were purchased from GeneTex (Irvine, CA, USA), whereas mouse monoclonal antibodies of EGFR, c-Met, Her2, Her3, phosphorylated JNK (p-JNK) and rabbit polyclonal antibody of c-Src were from Santa Cruz Biotechnology, Inc. (Dallas, TX, USA). The Src kinase inhibitor dasatinib was from Tocris Bioscience (Bristol, UK). The glycolysis inhibitor 3-Bromopyruvate (3-BrP) was from Santa Cruz Biotechnology, Inc. (Dallas, TX, USA). 

### 2.3. RNA Interference and Ectopic Gene Expression

Hic-5 and GAPDH were transiently knocked down by 12–25 nM Hic-5 siRNA from Dharmacon (Lafayette, CO, USA) and GAPDH siRNA from Santa Cruz Biotechnology, Inc. (Dallas, TX, USA), respectively. The cells were transfected by Hic-5 or GAPDH siRNA using the DharmaFECT 4 transfection reagent (Dharmacon, Lafayette, CO, USA) in antibiotic-free complete medium for 48 h, according to the manufacture’s protocol. The depletions of Hic-5 and GAPDH were validated by Western blot and RT-PCR. The target sequence of Hic-5 and GAPDH siRNAs are pools of four and three effective antisense nucleotides, respectively, described in the data sheets of TGFβ1I1 (7041) siRNA and sc-35448 GAPDH siRNA(h). Hic-5 expression plasmid, HIC5 (TGFB1I1) (p-Hic-5), was purchased from OriGene Technologies, Inc. (Rockville, MD, USA) with tagged GFP.

### 2.4. Proliferation Assay 

A cell proliferation assay was performed by utilizing 3-[4,5-dimethylthiazol-2-yl]-2,5-diphenyl tetrazolium bromide (MTT) as a water-soluble yellow dye that is readily taken up by viable cells and reduced by the action of mitochondrial dehydrogenases. After appropriate treatments of the cells, the dye was released and dissolved by DMSO and the absorbances were measured under a 535 nm wavelength.

### 2.5. Transwell Migration Assay 

Cells were seeded on a 24-well transwell migration insert (Nalge Nunc International, Rochester, NY, USA) in a complete medium for 24 h. After appropriate treatments, cells that had migrated to the underside of the insert membrane were stained with 0.3% crystal violet. The cells on the topside of the insert membrane were rubbed with a cotton swab. The migrated cells on the underside were imaged using phase contrast microscopy with a 40× magnification. The quantitation of the migrated cell was performed by measuring the number of crystal-violet-stained cells, using ImageJ software (version 1.50 i).

### 2.6. Transwell Invasion Assay

Cells were seeded on a 24-well transwell migration insert (Nalge Nunc International, Rochester, NY, USA) coated with Matrigel (Corning, MA, USA) in complete medium for 24 h. After appropriate treatments, cells that had invaded to the underside of the insert membrane were stained with 0.3% crystal violet. The cells on the topside of the insert membrane were rubbed with a cotton swab. The invaded cells on the underside were imaged using phase contrast microscopy with a 40× magnification. The quantitation of the invaded cell was performed by measuring the number of crystal-violet-stained cells, using ImageJ software (version 1.50 i).

### 2.7. Animal Experiments

HuCCT1 cells (4 × 10^6^) were subcutaneously inoculated on both left and right flanks of SCID mice for one week. Subsequently, indicated drugs were applied around cell inoculation site on one flank for the indicated times, whereas a vehicle (0.2% DMSO) was applied around that of the other flank in the same mouse serving as a control. After appropriate treatments, mice were sacrificed and the size of the HuCCT1 tumor volume was compared between the indicated groups and processed for the indicated experiments. The tumor volume (cm^3^) was measured as V = 1/2 × (l × w^2^), where V—volume, l—length, w—width, assuming an ellipsoid tumor with π = 3 according to the xenograft protocol described previously [40,41,42,43]. The advantage of this formula is based on the fact that width (w) and length (l) are the two diameters which can be measured accurately by a vernier caliper in vivo and there is no need to approximate the tumor height (which could result in an error). The therapeutic index (TI) of dasatinib was calculated as LD50/ED50, where LD50 is the lethal dose of dasatinib for 50% of the mice tested whereas ED50 is the minimum effective dose of dasatinib for 50% of the mice. For the TI calculation, we combined the data presented in the paper (dasatinib 0.5–2.0 mg/g mouse) and those obtained from higher doses of dasatinib (3.5–4.5 mg/g mouse), which revealed a substantial mortality rate, and that from lower doses of dasatinib (0.2–0.4 mg/g mouse), which displayed a lower effect of tumor suppression. After extrapolation of the dose-dependent curve fitting to the Hill equation, LD50 was estimated to be 2.8 mg/g mouse (n = 4), whereas ED50 was about 0.58 mg/g mouse (n = 6). Thus, the therapeutic index of dasatinib was estimated to be about 4.83 [LD50/ED50 = 2.8/0.58]. During animal experiments, which were approved by the Institutional Animal Care and Use Committee at Tzu Chi hospital, regulations relevant to the care and use of laboratory animals were followed. 

### 2.8. Extraction of Tissue 

The animal tissues were homogenized and extracted by a Bioruptor (Bioruptor Plus, Seraing, Belgium), according to the manufacturer’s protocol. Briefly, 50 mg of tissue was added to 50 mg of protein extraction beads, which were sonicated for 15 cycles at 4 °C. Subsequently, the supernatants were centrifuged at 14,000 rpm to remove the remaining insoluble materials and stored at −80 °C. 

### 2.9. Western Blots 

Western blots were performed according to our previous studies [33]. Briefly, the cells were lysed in a buffer containing 50 mmol/L Tris at pH 7.4, 50 mmol/L NaCl, 0.1% Triton X-100, 0.1% SDS, 0.3 mmol/L sodium orthovanadate, 50 mmol/L NaF, 1 mmol/L dithiotheritol, 10 μg/mL leupeptin and 5 μg/mL aprotinin. Proteins were separated and transferred to polyvinylidene difluoride membranes. The membranes were blocked in 20 mmol/L Tris (pH 7.6) and 250 mmol/L NaCl containing 5% dry milk and probed with antibodies against the indicated molecules. The band intensities on the blots were quantified using ImageJ software (version 1.50 i).

### 2.10. Immunohistochemistry (IHC) 

IHC was performed by the EnVision+ Dual Link System-HRP (DAKO, Carpinteria, CA, USA), a two-step staining technique using an HRP labelled polymer conjugated with secondary antibodies. Briefly, the tissue section was incubated with Dual Endogenous Enzyme Block to remove any endogenous peroxidase activity. Subsequently, the sample was incubated with primary Ab for 30 min, followed by the 2nd Ab-HRP labeled polymer for 30 min. Staining was completed by a 5–10 min incubation with 3,3′-diaminobenzidine (DAB+) substrate chromogen, which resulted in a brown-colored precipitate at the antigen site.

### 2.11. Statistical Analysis 

An ANOVA test was conducted to analyze the intensity differences between samples on the Western blot, IHC, cell invasion and the size differences between the tumors developed on mice skins under the indicated experimental conditions. Quantitative data were expressed as mean ± coefficient variation (C.V.).

## 3. Results

### 3.1. Enhanced Inhibition of HuCCT1 Cell Invasion by Cotargeting Hic-5 and Src 

**Figure 1:**
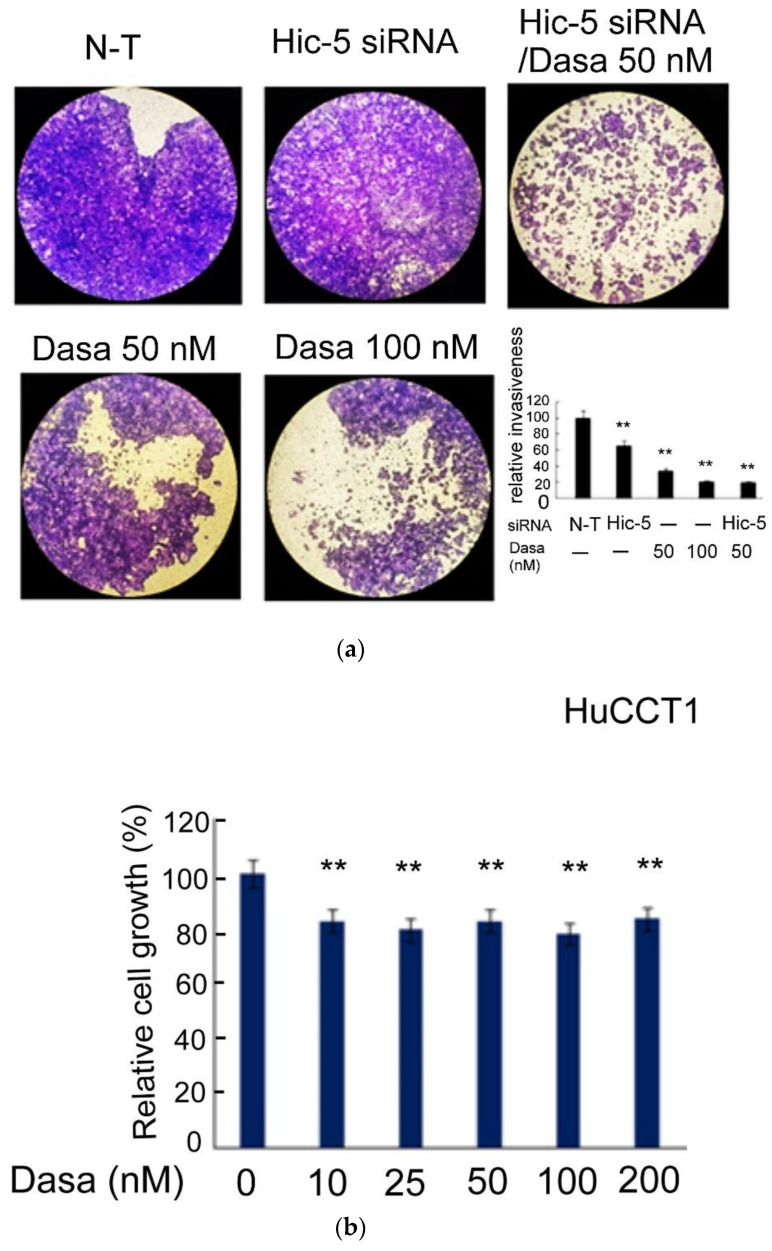
Src–Hic-5 signal cascade is required for HuCCT1 cell invasion. HuCCT1 cells were transfected with Hic-5 siRNA, treated with dasatinib (Dasa) or a combined transfection with Hic-5 siRNA coupled with treatment of Dasa at indicated concentrations for 48 h (**a**); HuCCT1 cells were treated with Dasa at indicated concentrations for 48 h (**b**). Transwell Matrigel invasion assay (**a**) and MTT assay (**b**) were performed. In (**a**), the images shown are the crystal-violet-stained invaded cell, pictured under a 40× magnification. Quantitation of the staining intensity using imageJ software is shown in lower right panel. Relative invasiveness was calculated taking the data of nontargeting (N-T) group as 100%. In (**b**), relative cell growth was calculated based on the absorbance measured under 535 nm, taking the data of Dasa at 0 nM as 100%. (**) represents the statistical significance (*p* < 0.005, N = 6) of the differences between the indicated sample and the N-T (**a**) and Dasa at 0 nM (**b**) groups, respectively.

In a previous report, we demonstrated cotargeting Src and Hic-5 using the Src inhibitor dasatinib and Hic-5 siRNA, respectively, suppressed the cell migration of HuCCT1 in an additive manner [33]. We further investigated whether the same effect could be observed in HuCCT1 cell invasion. As shown in the transwell Matrigel invasion assay (Figure 1a), dasatinib (50–100 nM) suppressed HuCCT1 cell invasion by 67~80% dose-dependently. Moreover, the depletion of Hic-5 by 25 nM Hic-5siRNA suppressed HuCCT1 cell invasion by 37%. Remarkably, Hic-5 siRNA coupled with 50 nM dasatinib dramatically decreased HuCCT1 cell invasion by 82%, an effect higher than a single treatment of Hic-5 siRNA (by 35%) or 50 nM dasatinib (by 67%) and close to that of 100 nM dasatinib (by 80%) (Figure 1a). This demonstrated an enhanced effect of knocking down Hic-5 coupled with an inhibition of Src activity on suppressing HuCCT1 cell invasion. To evaluate the possibility that the decrease of cell invasion triggered by Src inhibitor and/or Hic-5 siRNA resulted from the decrease of cell proliferation, an MTT assay in HuCCT1 cells treated by dasatinib or transfected with Hic-5 siRNA was performed. As demonstrated in Figure 1b, HuCCT1 cell growth was slightly and constantly suppressed by dasatinib by 20% across the concentration range of 10~200 nM, whereas the depletion of Hic-5 did not affect HuCCT1 cell growth). Taken together, it can be estimated that 50–100 nM dasatinib inhibited cell invasion by 48~64%, after excluding the amount of decreased cell growth due to the dasatinib treatment. 

**Figure 2:**
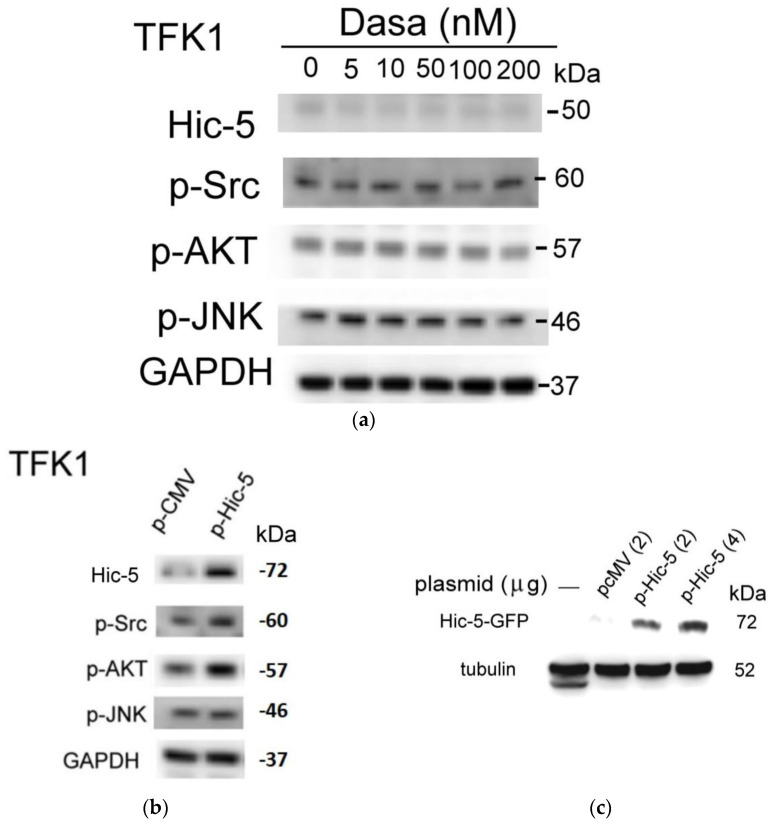

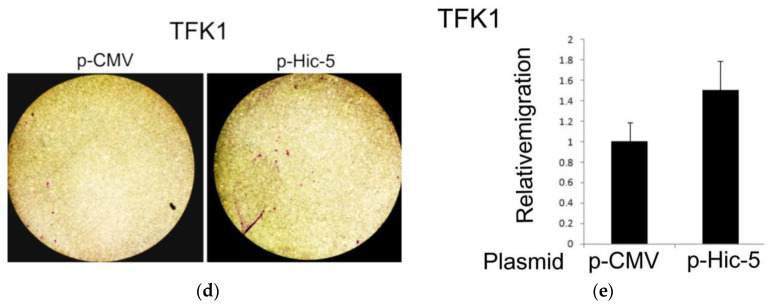
Ectopic expression of Hic-5 significantly enhanced the activation of Src, AKT and JNK for the cell migration of TFK1. TFK1 cells were treated with dasatinib (Dasa) at indicated concentrations (**a**) or transfected with Hic-5 expression plasmid (p-Hic-5) (**b**–**d**). Western blot of indicated molecules (**a**–**c**) and transwell migration assay of TFK1 (**d**) were performed. In (**b**,**c**), the Abs used for detecting p-Hic-5 encoded protein (Hic-5–GFP fusion protein) were Hic-5 and GFP, respectively, revealing the same molecular weight. In (**d**), the crystal-violet-stained, migrated cells were pictured under 40× magnification. (**e**) is the quantitative data of (**d**) using ImageJ software. Relative motility was calculated taking the data of the p-CMV vector as 1.0. The data shown are representative of two reproducible results.

To strengthen the notion that Src–Hic5 signaling is essential for the progression of CCA, another conventional CCA cell line, TFK1, was employed. TFK-1 was established from a surgical tumor of extrahepatic bile duct carcinomas, histologically diagnosed as partly papillary adenocarcinoma and partly differentiated tubular adenocarcinoma. However, Hic-5 mRNA and protein (Figure 2a) were very barely detected in TFK1, consistent with the very low motility of TFK1 cells observed in the transwell migration assay (Figure 2c, p-CMV group). These were in contrast with those observed in the high Hic-5 expressing HuCCT1 cells with high motility [33]. Moreover, dasatinib (5–200 nM) could not suppress the activation of Src, AKT and JNK in TFK1 cells (Figure 2a). Interestingly, the ectopic expression of Hic-5 (using Hic-5 expression plasmid driven by CMV promoter, p-Hic-5, 1.0 μg/mL) significantly activated Src and AKT (Figure 2b) compared with that of the p-CMV vector group. However, JNK was not activated by p-Hic-5. Notably, p-Hic-5 contains a GFP sequence added to the 3′ of the Hic-5 coding region as a tag for validating the ectopic expression of the Hic-5–GFP fusion protein (MW: 72 kDa) by the Western blot of GFP. As demonstrated in Figure 2c, whereas Hic-5–GFP could not be detected in the parental TFK1 cells (without plasmid transfection) and TFK1 cells transfected with the p-CMV vector, it was increased in TFK1 cells transfected with 2–4 μg/mL p-Hic-5, dose-dependently. Nonetheless, p-Hic-5 slightly increased the cell migration of TFK1 by 1.3–1.5-fold only (Figure 2d,e), suggesting that the increased Hic-5–GFP fusion protein expression per se was not enough to fully elevate TFK1 cell motility, probably due to the low efficiency of the Hic-5–GFP fusion protein in fully elevating the Src–AKT–JNK signaling. In addition, dasatinib did not decrease the cell growth of TFK1 cells using an MTT assay.

### 3.2. Dasatinib Suppressed HuCCT Cell Progression In Vivo

**Figure 3:**
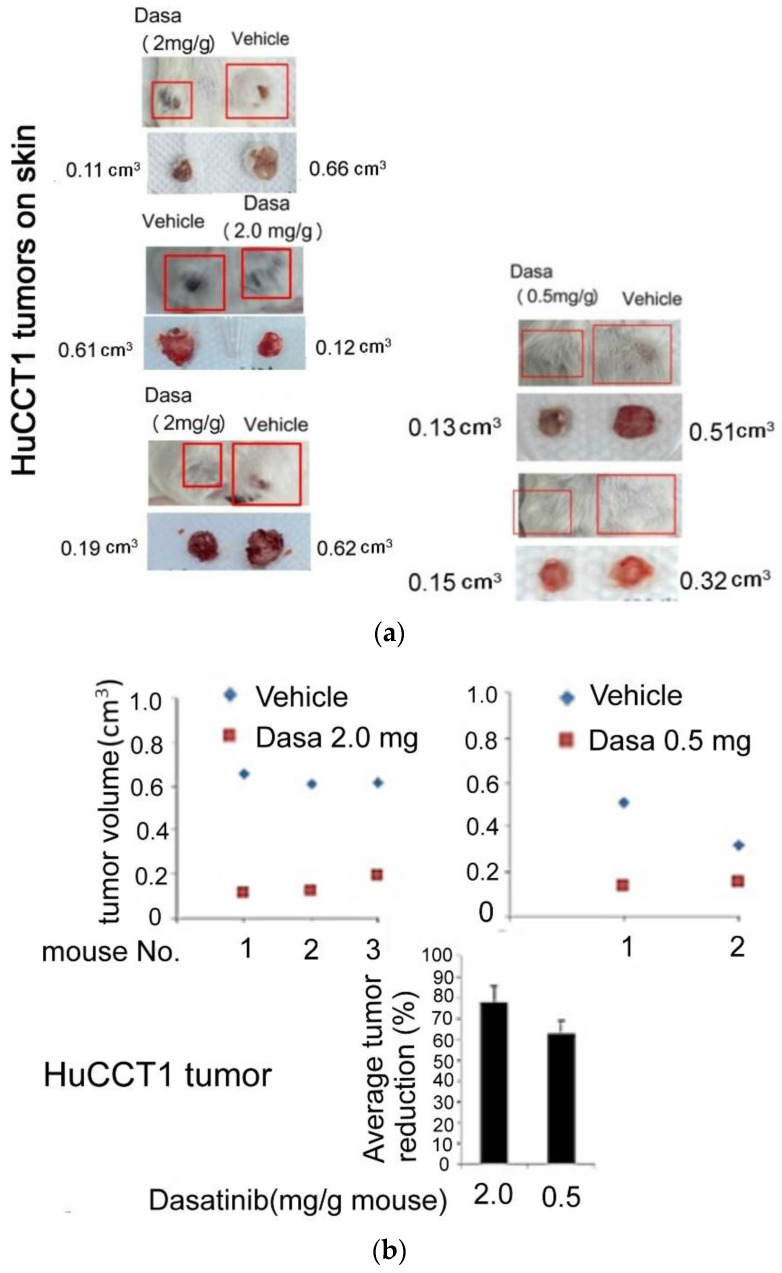

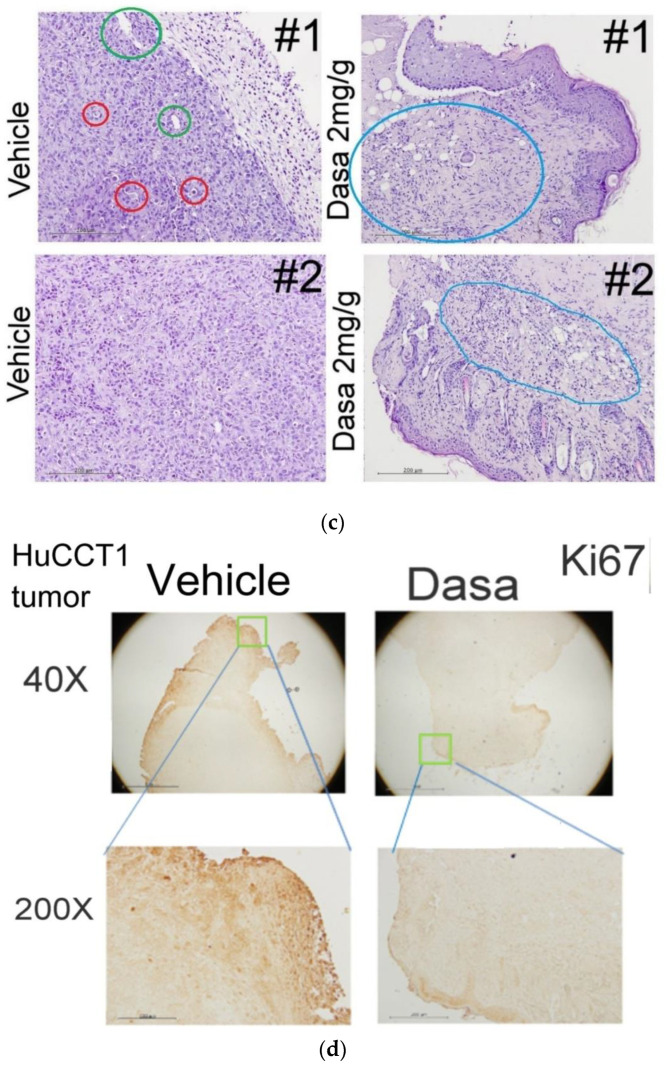
Dasatinib suppressed HuCCT1 tumor development on SCID mice. HuCCT1 cells (4 × 10^6^) were subcutaneously inoculated on both left and right flanks of three SCID mice for 1 week, followed by applying dasatinib (2 mg/g mouse) (**a**, left panel), or dasatinib (0.5 mg/g mouse) (**a**, right panel) around the cell inoculation site on one flank twice a week whereas 0.2% DMSO (vehicle) was applied around that on the other flank in the same mouse serving as a control. After drug injection for 1.5 (**a**, left panel for 2 mg Dasa/g mouse) and 1.0 (**a**, right panel for 0.5 mg Dasa/g mouse) months, the HuCCT1 tumors were collected and pictured (**a**) and then processed for H.E. staining (**c**) and IHC of Ki-60 (**d**). (**b**) is the quantitative data for (**a**), showing the average reduction rate for each Dasa concentration (N = 3 and N = 2 for 2.0 and 0.5 mg Dasa/g mouse, respectively). In (**a**,**b**), the size of tumors was designated as the volume (cm^3^) calculated as described in Materials and Methods. In (**c**), the black numbers indicate two different spots (denoted as #1 and #2) on the tissue sections from HuCCT1 tumors with indicated treatments selected for photography. The red and green circles enclose the duct formation and vein, respectively, in the tissues from the vehicle-treated group (left panel) whereas blue circles enclose the granulation tissue, fibrotic tissue and inflammation cells across the tissues from the dasatinib (2 mg/g mouse) group (right panel). In (**d**), the brown staining regions indicate the locations of Ki67. The staining sections were photographed under 40- (upper panel) and 200- (lower panel) fold magnification. The images in the lower panels show the magnified regions selected from marked insets (green rectangles) in the upper panel. The intensity was quantitated by Image J. software.

To validate whether Src–Hic-5 signaling is essential for HuCCT1 cell progression in vivo, we investigated the effect of dasatinib on preventing the development of an HuCCT1 tumor in SCID mice. HuCCT1 cells (4 × 10^6^) were subcutaneously inoculated on both left and right flanks of SCID mice for 1 week. Subsequently, dasatinib (0.5 or 2 mg/g mouse) was applied around the cell inoculation site on one flank twice a week while a vehicle (0.2% DMSO) was applied on the other flank in the same mouse serving as a control. In the 2 mg/g mouse dasatinib group, the mice exhibited a prominent reduction of the HuCCT1 tumor, and three of them are demonstrated in Figure 3a (left panel). After dasatinib injection for 1.5 months, the volume of tumors developed on one flank of each of the mice treated with 2 mg/g dasatinib were about 0.11, 0.12 and 0.19 cm^3^, whereas those of the paired vehicle-treated group in the same mouse were about 0.66, 0.61 and 0.62 cm^3^, respectively. This revealed a reduction of about 83.4, 80.4 and 70%, respectively, (average 78%) of the HuCCT1 tumor development on mice skins treated with 2 mg/g dasatinib compared with each of the vehicle-treated groups (Figure 3b, upper left and lower panel). In the 0.5 mg/g mouse group, the reduction of tumor volume was less evident, and two of them are demonstrated in Figure 3b (right panel). After dasatinib injection for 1.0 month, the tumor developed on skins of the mice treated with 0.5 mg/g dasatinib were 0.13 and 0.15 cm^3^, whereas those of the paired vehicle-treated group in the same mouse were 0.51 and 0.32 cm^3^, respectively. This revealed a reduction of about 74 and 51%, respectively, (average 62.5%) of the HuCCT1 tumor development on mice skins treated with 0.5 mg/g dasatinib, compared with each of the vehicle-treated groups (Figure 3b, upper right and lower panel). The therapeutic index of dasatinib, calculated by (LD50/ED50), was estimated to be about 4.83, where LD50 (the lethal dose of dasatinib for 50% of the mice tested) was 2.8 mg/g mouse and ED50 (the minimum effective dose of dasatinib for 50% of the mice) was 0.58 mg/g mouse (please see detailed calculation process in Section 2.7). Collectively, dasatinib dose-dependently suppressed the progression of the HuCCT1 tumor. To examine the patho-physiological changes associated with the suppression of the HuCCT1 tumor development triggered by dasatinib, a hematoxylin–eosin (H.E.) stain of the tissue sections was performed to observe the status of the tumor progression, whereas the immunohistochemistry (IHC) of Ki 67 was used for assaying the proliferative activities of the tumors. As shown on two different spots in the vehicle-treated tumor sections (Figure 3c, left panel), polymorphic, crowd and poorly differentiated HuCCT1 tumor cells with a high nucleus-to-cytoplasm ratio were observed. Moreover, the tissues exhibited scant ducts formation (red circles) and small blood vessels (green circles) with some invasive tumor cells inside. In contrast, on two different spots in the tissue sections from dasatinib-treated mice (2 mg/g mouse), no residual tumor cell was observed. Instead, it presented as granulation and fibrotic tissue with chronic inflammation across the sections (blue circles). On the other hand, the immunostaining of Ki67 demonstrated the expression of Ki-67 was reduced in two of the 2.0 mg/g dasatinib-treated tissue sections by about 20–30% compared with that in the other two vehicle-treated group (Figure 3d), indicating the proliferative activities of HuCCT1 tumors were significantly suppressed by dasatinib. This is consistent with the slightly decreased cell growth (by 20%) of cultured HuCCT1 cells induced by dasatinib for 48 h using the MTT assay (Figure 1b). Taken together, the inhibition of Src activity could suppress the development of HuCCT1 tumors on SCID mice skin. 

### 3.3. Dasatinib Suppressed Src/Hic-5/AKT/JNK Signaling In Vivo

**Figure 4:**
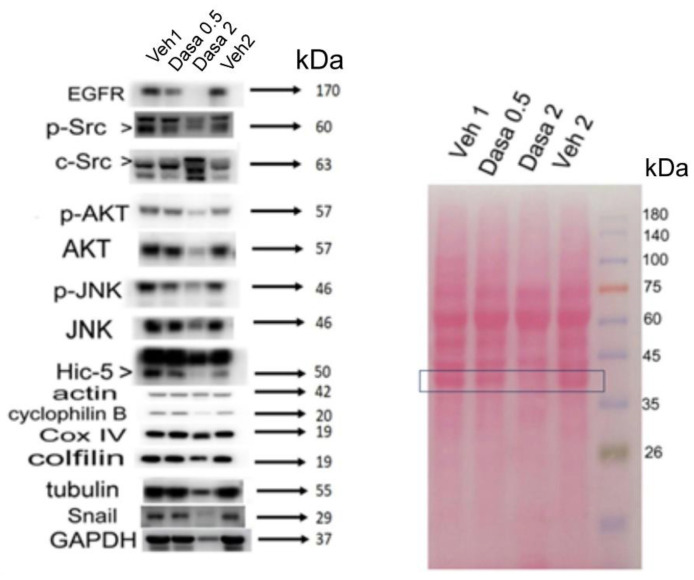
Signal molecules suppressed in HuCCT1 tumors treated with dasatinib. The tissue lysates of HuCCT1 tumors harvested from SCID mice skins treated with vehicles (Veh1 and Veh2) and dasatinib at indicated concentration (Dasa 0.5 and Dasa 2 mg/g mouse, paired with Veh1 and Veh2, respectively) were extracted for a Western blot of the indicated molecules (left panel). Ponceau S staining of the blot (right panel) was demonstrated as a loading control. In the right panel, blue rectangles enclose the band close to 37 kDa, suspected of GAPDH, which was shown to be decreased in dasatinib (2.0 mg/g)-treated samples in a Western blot. The data shown are representatives of two reproducible results.

To investigate whether HuCCT1 cell progression suppressed by dasatinib in vivo was associated with the decrease of Src–Hic-5–AKT–JNK signaling as has been observed in vitro [33], the relevant molecules in tumors from dasatinib and the paired vehicle-treated mice were compared. As expected, Hic-5 expression and Src/JNK activity (p-Src and p-JNK) were decreased in the HuCCT1 tumor treated with both 0.5 mg/g and 2.0 mg/g mouse of dasatinib by 80–90% compared with those treated with the vehicle (0.2% DMSO) (vehicle 1 and vehicle 2, respectively) in each of the same mice (Figure 4, left panel). Moreover, the AKT activity (p-AKT) was greatly decreased in 2.0 mg/g dasatinib-treated tumor by 85% and slightly decreased in 0.5 mg/g dasatinib-treated group by 10% (Figure 4, left panel). Moreover, the unphosphorylated JNK and AKT were also downregulated in the HuCCT1 tumor treated with dasatinib at the doses of 0.5 mg/g and 2.0 mg/g by 20–25 and 80–90%, respectively. However, the unphosphorylated Src (c-Src, with a lower molecular shift in the 2.0 mg/g sample) was not decreased and even slightly elevated in both dasatinib-treated tumors, probably due to a positive feedback response to dasatinib. These results suggested that a long-term treatment of high-dose Src inhibitor disrupted the downstream signaling of Src not only by decreasing enzyme activities but also protein levels of the relevant signal molecules. Importantly, this implicated that Hic-5 coupled with Src, AKT and JNK were within the same signal cascade in HuCCT1 tumors as was established in cultured HuCCT1 cells [33]. In addition, both phosphorylated and unphosphorylated EGFR, the oncogenic RTK upstream of Src [44], were also greatly decreased in dasatinib-treated HuCCT1 tumors in a dose-dependent manner (Figure 4, left panel), suggesting EGFR positively cross-talked with Src in HuCCT1 tumors, thus could also be suppressed by dasatinib. Moreover, Snail (SNA), the well-known EMT transcriptional factor downstream of RTK and Hic-5 [35], was also abolished in the 2.0 mg/g dasatinib-treated HuCCT1 tumor (Figure 4, left panel). Together, the metastatic signaling molecules including those in the Hic-5–Src–AKT–JNK cascade and upstream EGFR could be suppressed by a long-term treatment of Src inhibitor, which was closely associated with the suppression of HuCCT1 cell progression on SCID mice skin. 

### 3.4. Long-Term High-Dose Dasatinib Treatment Suppressed Metabolic Enzyme GAPDH and Cytoskeletal Protein Markers

In addition to the decrease of metastatic signal molecules, we surprisingly found several conventionally used internal control molecules including GAPDH, tubulin, cyclophilin B and cofilin were also dramatically suppressed (by 85–95%) in the high-dose (2.0 mg/g mouse) dasatinib-treated HuCCT1 tumor, whereas COX IV and β-actin were suppressed to a much lesser extent (by 25 and 8.0%, respectively). On the other hand, all these markers were not decreased in the HuCCT1 tumor treated with a lower dose of dasatinib (0.5 mg/g mouse) and could serve as internal controls under this condition. To exclude the possibility of a protein loading error, we examined the Ponceau S staining of the Western blots to compare the protein amounts loaded between samples. As shown in Figure 4 (right panel), the bands in the Ponceau S staining were consistent between samples in general. However, in the high-dose (2.0 mg/g mouse) dasatinib-treated sample, the protein around 37 kDa was prominently decreased, compared with that in the vehicle groups (see blue rectangle). Notably, this alteration was consistent with that observed in the Western blot, i.e., a greatly decreased GAPDH (M.W. 37 kDa) in high-dose (2.0 mg/g) dasatinib-treated tumor (Figure 4, left panel). It is worth noticing that in the tumors treated with a high dose of dasatinib (2.0 mg/g), some of the protein markers (GAPDH, tubulin, cyclophilin B, cofilin) were suppressed dramatically (more than 85%) whereas the others (β-actin and COX IV) were decreased to a lesser extent (12 to 25%, respectively). Actually, in the past decades, the protein markers that were highly suppressed by dasatinib including GAPDH [45,46,47], tubulin [38,48], cyclophilin B [49,50,51] and cofilin [39,52,53] have been found to be closely associated with tumor metastasis. It is very probable that these proteins may also be involved in Src-mediated HuCCT1 cell progression; among them, GAPDH, which was the marker decreased the most by 2.0 mg/g dasatinib (by 95%) (Figure 4, left panel), is interesting and highlighted. 

### 3.5. GAPDH Is a Downstream Effector Mediating Hic-5–Src–AKT Signaling for HuCCT1 Development

**Figure 5:**
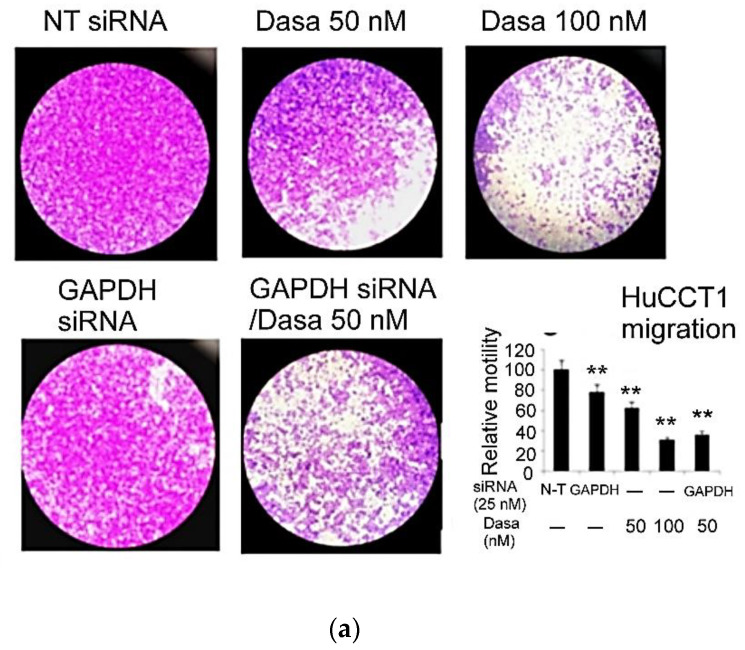

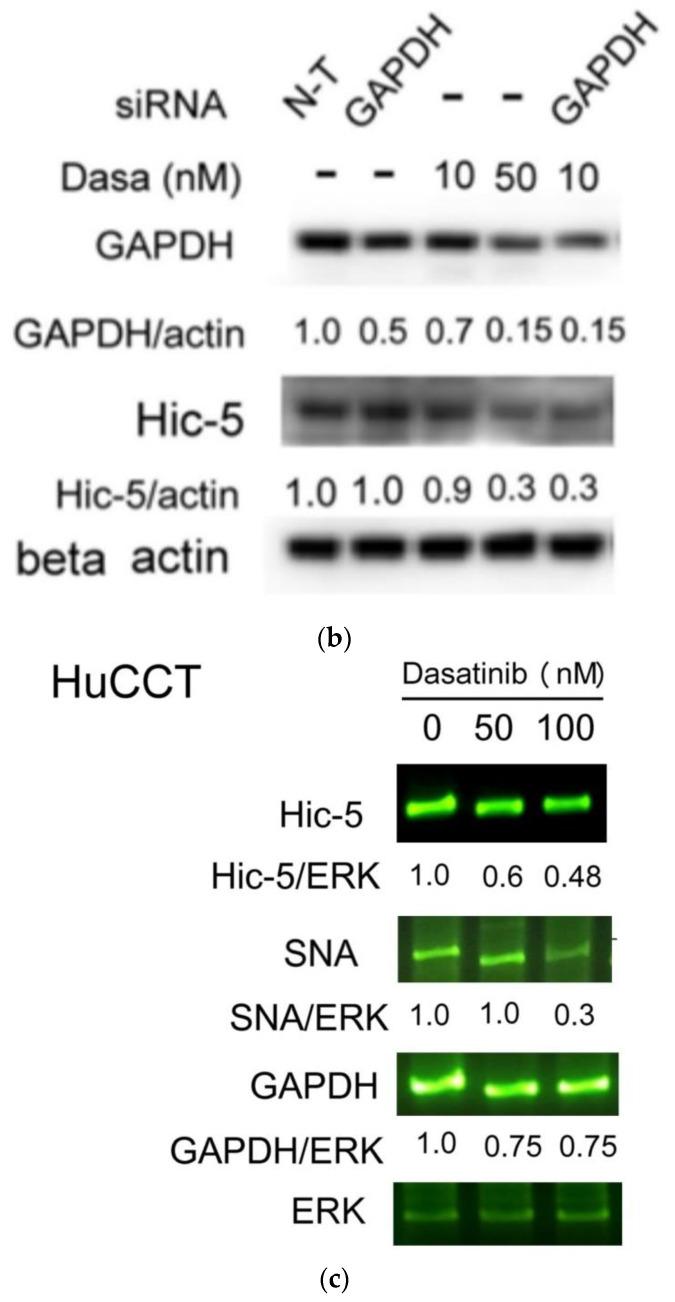
GAPDH mRNA and protein are downregulated by dasatinib and required for HuCCT1 migration. HuCCT1 cells were transfected with GAPDH siRNA and/or treated with the Src inhibitor dasatinib (Dasa) at indicated concentrations (**a**,**b**) or treated with dasatinib at indicated concentrations (**c**) for 48 h. Transwell migration assay (**a**), Western blot (**b**) and RT-PCR (**c**) were performed using β actin (**b**) and ERK (**c**) as internal controls. In (**a**), the images are the crystal-violet-stained, migrated cells pictured under a 40× magnification. The resultant quantitative data obtained using ImageJ software are shown in lower right panel. Relative motility was calculated taking the data of the nontargeting (N-T) group as 100%. (**) represents the statistical significance (*p* < 0.05, N = 3) of the difference between the indicated samples and the nontargeting (N-T) group. In (**b**,**c**), the numbers below each of the bands indicate the relative band intensities, taking the data of β actin (**b**) and ERK (**c**) as 1.0. The data shown are the average of two reproducible results, with a coefficient of variation (C.V.) of 9%.

Classically, GAPDH is an essential enzyme catalyzing the redox reaction in the glycolytic pathway by converting glyceraldehyde-3-phosphate to 1,3-bisphosphoglycerate with a reduction of NAD^+^ to NADH. Recently, it was found to play an important role in tumorigenesis associated with enhanced glycolysis and energy production required for cancer progression, i.e., the Warburg effect [46]. Moreover, GAPDH expression was elevated in colon cancer and metastatic liver tissues [46], which suggested that an increased GAPDH contributed to colon cancer metastasis by providing more energy required for tumor progression. Remarkably, the depletion of the metastatic scaffolding protein NEDD9, which reduced Src activity could lead to the reduction of glycolytic enzymes including GAPDH [47]. Moreover, decreasing GAPDH caused a downregulation of the gene expression of Snail (SNA), one of the well-known EMT transcription factors involved in tumor progression [45]. Interestingly, SNA was also suppressed in the high-dose (2.0 mg/g mouse) dasatinib-treated HuCCT1 tumor (Figure 4). Accordingly, we sought to investigate whether GAPDH was involved in cell migration mediated by Hic-5–Src signaling in cultured HuCCT1 cells. As demonstrated in Figure 5a, the depletion of GAPDH (by GAPDH siRNA) for 48 h suppressed HuCCT1 cell migration by 23%, compared with the nontargeting (N-T) group, whereas 50 and 100 nM dasatinib suppressed HuCCT1 cell migration by 40 and 68%, respectively. Moreover, the combined inhibition of Src (using Dasa at 50 nM) coupled with the depletion of GAPDH decreased HuCCT1 cell migration by 75%, an extent significantly higher than that observed in either the depletion of GAPDH (by 23%) or the 50 nM Dasa-treated group alone (by 38%) (Figure 5a). The efficiency of GAPDH siRNA was verified in Figure 5b, showing a decrease of GAPDH protein by 50% in cells transfected with 25 nM GAPDH siRNA for 48 h compared with that of the nontargeting (N-T) group. Interestingly, the expression of GAPDH protein was decreased by dasatinib at 10 and 50 nM for 48 h by 30 and 85%, respectively, using β-actin as the loading control (Figure 5b). Such an inhibitory effect was not observed in HuCCT1 cells treated with dasatinib for 24 h in our previous study [33]. Moreover, 10 nM dasatinib coupled with GAPDH siRNA decreased the expression of GAPDH by 85%, an extent significantly higher than either GAPDH siRNA (by 50%) or 10 nM dasatinib alone (by 30%) (Figure 5b). Moreover, consistent with the data demonstrated in our previous report [33], 10 and 50 nM dasatinib inhibited the expression of Hic-5 by 10 and 70%, respectively, (Figure 5b). Surprisingly, 10 nM dasatinib combined with GAPDH siRNA decreased the expression of Hic-5 by 70%, much higher than 10 nM dasatinib alone (10%), implicating GAPDH coupled with Src can also be upstream of Hic-5. On the other hand, the RT-PCR analysis demonstrated dasatinib significantly suppressed the mRNA of GAPDH and Hic-5 by 25–40% at 50 and 100 nM concentration in a dose-dependent manner, whereas it suppressed SNA mRNA at 100 nM by 70% for 48 h (Figure 5c). In contrast, dasatinib did not decrease the mRNA of ERK, one of the MAPK, which may serve as an internal control (Figure 5c). Taken together, GAPDH played as a downstream effector in Src–Hic-5 signaling cascade for HuCCT1 cell migration.

### 3.6. Suppression of HuCCT1 Cell Migration by Glycolysis Inhibitor 3-Bromopyruvate 

**Figure 6:**
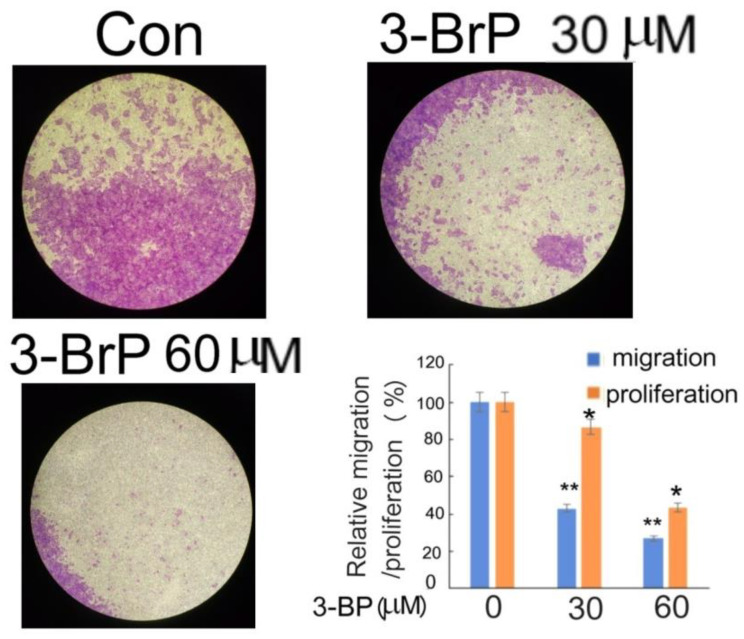
Glycolysis inhibitor 3-Bromopyruvate suppressed HuCCT1 progression. HuCCT1 cells were treated with 3-Bromopyruvate (3-BrP) at indicated concentration for 48 h using the sample of untreated cells (0 μM 3-BrP) as controls (Con). Transwell migration assay and cell proliferation assay were performed. On the upper and left panels, the images of migration assay are the crystal-violet-stained, migrated cells pictured under a 40× magnification. Cell proliferation was analyzed using the MTT assay described in Materials and Methods. The resultant quantitative data of migration (blue bars), using ImageJ software, and cell proliferation (orange bars) assay are shown in the lower right panel. Relative motility and cell proliferation were calculated taking the data of the control (0 μM 3-BrP) group as 100%. (**) and (*) represent the statistical significance (*p* < 0.005 and 0.05, respectively, N = 4) of the difference between the indicated samples and the control.

Since GAPDH is required for one of the key steps in the glycolysis pathway, we further investigated whether glycolysis was required for HuCCT1 cell progression. To this end, 3-Bromopyruvated (3-BrP), one of the anticancer agents capable of targeting the glycolytic enzymes, including GAPDH [46,54], was employed. As demonstrated in Figure 6, 30 and 60 μM 3-BrP effectively suppressed HuCCT1 cell migration by 62 and 75% in a dose-dependent manner, whereas the MTT assay demonstrated the inhibition of HuCCT1 cell growth, by 12 and 65%, respectively. Remarkably, while 30 μM 3-BrP slightly reduced HuCCT1cell growth (by 12%), it induced a much higher inhibitory effect on HuCCT1 cell migration (by 62%). However, 60 μM 3-BrP exerted dramatic suppressive effects on both the proliferation and migration (65 and 75%, respectively) of HuCCT1 cells, revealing a prominent toxic response of HuCCT1 cells at this concentration. In addition, 15 μM 3-BrP did not exhibit a suppressive effect on both the migration and proliferation of HuCCT1 cells. In summary, 30 μM was the most optimal concentration for 3-BrP to trigger a substantially suppressive effect on HuCCT1 cell migration by about 50%, after excluding the slightly inhibitory effect on cell growth. 

## 4. Discussion

### 4.1. Targeted Therapy Aiming at Src–Hic-5–GAPDH Cascade for Preventing CCA Progression

Previously, although the role of focal adhesion signaling molecules including FAK [55] and Src [33] in CCA migration and invasion have been demonstrated in vitro, whether they are really essential for CCA progression has not been demonstrated in vivo. In our studies, we found the Src inhibitor dasatinib not only effectively suppressed HuCCT1 cell migration [33] and invasion (Figure 1a) in a cultured system but also suppressed the HuCCT1 tumor development on SCID mice after a long-term treatment of dasatinib (Figure 3). Dasatinib is a potent inhibitor of multiple tyrosine kinases capable of inhibiting ABL and SRC, c-KIT, PDGFR-α, PDGFR-β and ephrin receptor kinases [56]. Previously, dasatinib was used for the treatment of CML and AML with Philadelphia chromosome-positive (Ph+) via inhibiting Src-Abl1 kinase, a deregulated chimeric kinase responsible for both diseases [57]. Dasatinib also inhibited the growth of patient-derived tumor in mice by targeting LIMK1 [58]. In this study, we found dasatinib suppressed not only the Hic-5/Src/AKT/JNK signaling but also the expression of other metastasis-related molecules including EGFR, tubulin and GAPDH in HuCCT1 tumors (Figure 4). Thus, dasatinib appears to be a promising drug capable of preventing the progression of CCAs with an increased Src activity. However, in previous therapeutic managements, the risk of side effects caused by dasatinib was very high [59,60,61]. These included fluid retention, pleural effusions, body aches, tingling, leukopenia, diarrhea and congestive heart failure. In our studies, we found the concentration of dasatinib used for the effective suppression of HuCCT1 cell migration [33] and invasion (Figure 1a) could be lowered when it was combined with Hic-5 siRNA. A similar phenomenon can also be observed in migration assay using a combination of dasatinib with GAPDH siRNA (Figure 5a). Therefore, it is worth validating whether an in vivo knockdown of Hic-5 and/or GAPDH may lower the concentration of dasatinib used for effectively preventing CCA progression in future preclinical and clinical trials.

### 4.2. The Potential Role of GAPDH in Src–Hic-5 Signal Cascade in HuCCT1

In the animal experiments, the dramatic reduction of some conventionally used protein markers including GAPDH, tubulin, cyclophilin B and cofilin in CCA progression was unexpectedly found in HuCCT1 tumors treated with a high dose of dasatinib (Figure 4). As mentioned above, these protein markers were known to be closely associated with tumor progression. For example, elevated tubulin was associated with cancer progression and chemotherapy resistance of ovarian cancer, breast cancer, gastric cancer and lung cancer [48], which can be targeted by paclitaxel [38]. Moreover, cyclophilin B overexpressed in various malignant tumors and could be an independent prognostic indicator of colon cancer [49,50,51]. In addition, cofilin-1 was identified as a novel mediator for the metastatic potentials and chemoresistance of prostate cancer cells [53].

Among the protein markers, GAPDH, which was dramatically abolished in HuCCT1 tumors treated with dasatinib (Figure 4), was the most potentially involved in HuCCT1 cell progression. Although GAPDH has been known as a housekeeping enzyme and thus commonly used as an internal marker in Western blotting and RT-PCR analyses, mounting evidence suggests that GAPDH, also called a moonlighting protein, may play nonenzymatic roles with diverse functions according to subcellular distributions. Apart from its classical role in energy production in mitochondria, membrane-bound GAPDH is required for endocytosis and iron transport. Whereas cytoplasmic GAPDH regulates mRNA stability, nuclear GAPDH is involved in the transcriptional gene regulation and maintenance of DNA integrity [62]. More importantly, a significant role of GAPDH in the progression of colon cancer, liver cancer [45] and lung cancer [47] has been established. In the present study, we found GAPDH was involved in progression of HuCCT1 cells mediated by Src both in vitro and in vivo (Figure 4 and Figure 5). Accordingly, there are several mechanisms proposed to explain the possible role of GAPDH in Src-mediated CCA progression. First, the glycolytic energy produced via the Src–GAPDH pathway required for increasing metabolism during CCA progression may be critical. Previously, Src contributed to the Warburg phenotype by inactivating the pyruvate dehydrogenase (PDH) complex via tyrosine phosphorylation, and this metabolic effect was essential for Src-driven malignancy and therapy resistance [63]. Moreover, targeting Src attenuated lactate dehydrogenase (LDH) activity leading to a reduction of invasiveness in head and neck cancer and breast cancer [64]. Thus, it is very probable that Src-triggered CCA progression may also be achieved via enhancing glycolytic energy production by increasing GAPDH activation and expression. This notion can be supported by our results showing the Src inhibitor dasatinib suppressed GAPDH expression both in vitro and in vivo (Figure 4 and Figure 5b, respectively) whereas the depletion of GAPDH may enhance the suppressive effect of dasatinib on the cell migration of HuCCT1 (Figure 5a). Moreover, 3-BrP, one of the potent inhibitors of glycolysis may also effectively suppress HuCCT1 cell migration with a slight inhibition of cell growth at a 30μM concentration (Figure 6). These suggested the energy production from glycolysis is required for Src- and Hic-5- triggered CCA progression. It is also worth noting that the effect of dasatinib on suppressing GAPDH appears to be a long-term response. This was evidenced by the dramatic GAPDH suppression in dasatinib-treated HuCCT1 tumors on mice skin for 2 months (Figure 4) and a significant suppression of GAPDH expression in cultured HuCCT1 treated with dasatinib for 48 h (Figure 5b) but not for 24 h in our previous study [33]. Second, GAPDH may play as one of the effector molecules in the signaling pathway mediated by Src. This can be implied by the fact that the Src-mediated phosphorylation of GAPDH regulates its nuclear localization and cellular response to DNA damage [65]. In addition, it has been suggested that the putative binding partners of the proline-rich GAPDHS motif relies on the SH3 domain-binding protein 4, which is often contained within signaling kinases, including Src [66], suggesting a possible interaction between Src and GAPDH. The third mechanism involves the participation of GAPDH in the transcriptional regulation of SNA suggested by a previous report [45]. Since we found both GAPDH and SNA could be positively regulated via the Src pathway in HuCCT1 cells (Figure 4 and Figure 5c), it is probable that GAPDH plays a role in mediating the Src-triggered transcriptional regulation of SNA. This issue can be addressed by observing whether GAPDH can bind to the SNA promoter region in HuCCT1 cells. Taken together, it is worth investigating the detailed mechanisms responsible for GAPDH mediating the Src–Hic-5 signaling during HuCCT1 cell progression. 

## 5. Conclusions and Perspective

Using in vitro and in vivo models, we demonstrated the Src–Hic-5–AKT–JNK pathway was essential for HuCCT1 cell progression, which is consistent with the indicated signaling deregulated in clinical samples [33]. Moreover, we found GAPDH also played a critical role in this pathway. In the future, it is tempting to investigate whether targeting Src coupled with Hic-5 and/or GAPDH is efficient for preventing CCA progression in future clinical trials.

## Data Availability

Not applicable; no data are available for supporting the results in this study.
